# Hospital Reports

**Published:** 1874-04

**Authors:** 


### Hospital Reports.

***Woman's Hospital of the State of Illinois.*** *Sub-Involution—Endometritis—Displacement.* By W. C. LYMAN, M.D., Assistant Surgeon to the Hospital.

In presenting the following abstract of cases treated at the Woman's Hospital, we wish to state that we have taken a number of average or type cases of several forms of disease peculiar to women, and those that were treated long and connectedly enough to arrive at some conclusion as to the length of time, the method, and the result of the treatment; leaving entirely out of consideration those cases that were under observation but a short time, and that were so irregular in attendance that no value can be placed upon the result, whatever it may have been.

We will first consider cases of sub-involution—five in number. Of these, the time that had elapsed subsequent to parturition varies from about three months to six years. The ages vary from twenty-three to thirty-five years; the average age, twenty-nine years. Length of time under treatment, from two to twenty months; average period, five months. Number of local applications, ranges from four to fifty-nine; average number, eighteen.

The method of treatment adopted in these cases was practically the same, and an abstract of the record on the hospital journal of one or more cases will sufficiently illustrate it.

Case I. Mrs. M.; age thirty; thirteen years married; has had two children, the youngest about four years of age; has never been well since last confinement; at the present time is generally debilitated, dyspeptic, and has palpitation of the heart on slight exertion; pain in loins; copious leucorrhœa; inability to walk, except very short distances; menstrual function regular each three weeks, occasionally copious, and always painful for the past three years.

Physical examination showed the womb low down in the pelvis, os externum patulous, whole cervix apparently increased in size, and copious tenacious mucus bathing all parts within the field of the speculum.

The sound showed the uterine cavity nearly normal in direction, but three and one-fourth inches in depth, the canal of the cervix wide, and the os internum easily passed. The retraction of the sound was followed by considerable mucus, streaked with blood; a granular condition could be observed just within the enlarged and dilated cervix.

The local treatment in the case was begun by the application of nitric acid to the interior of cervix and body of the organ, followed by the application of glycerine, and a cotton ball pessary saturated with glycerine, to remain in contact with the cervix till the following day. The application was attended by some pain, and a slight hemorrhage, both of which subsided without any special treatment except recumbency.

This patient was treated twice weekly for two weeks with a solution of tannin in glycerine, one part to two, applied to interior of cervix and body, and upon external parts of the cervix. Upon examination at that time, a perceptible diminution in size of the cervix, a diminished depth of the uterine cavity, and less mucous discharge, were to be observed.

Application was again made of the nitric acid by the same method as before, followed by a series of bi-weekly applications of Lugal's solution of iodine and glycerine for four weeks, omitting a week in which a menstrual period occurred, with less than the usual amount of pain, and less in quantity of leucorrhœa; in short, it approached her former natural and healthy periods in general character.

Then followed a third application of nitric acid, and another course of bi-weekly applications of the solution of tannin, or of iodine, or of tincture chloride of iron, as the appearance of the case seemed to demand at the time of application.

At the end of three weeks more, the patient had passed another period, regular as to time, quantity, and character.

The cervix appeared healthy, and the uterine cavity was restored to its natural depth, involution being completed by surgical aid.

We next come to consider twenty-six cases of endometritis. The inflammatory effects being apparent in the cervix and body of the uterus, and a small number (six) being attended by ulceration or erosion about the margin of the os externum, the disease being wholly within the cavity of cervix or body generally.

Of these, sixteen were married, and ten single. Their ages ranged from twenty-one to forty-three years; average age, thirty-one years. The number of applications ranged from six to forty-eight; average number, twenty-one. Period under treatment ranged from one to twenty months; average period, a fraction under four months.

To correctly set forth the method of treatment pursued in these cases, I will again quote from the hospital record:

Case 2. Mrs. S.; age forty-two years; number of pregnancies ten, of which six were abortions; inclined to hysteria; is thin, dyspeptic, and irritable; complains of pain in back in ovarian region and down the thighs, and upon sexual intercourse; is subject to frequent attacks of neuralgia of chest, head, face, and along spinal column.

Examination with speculum shows a glairy mucous discharge from the os uteri, with an areola of heightened color around it; a somewhat enlarged cervix.

Digital examination shows only a tenderness of cervix, with induration and hyperplasia.

The sound is passed only with pain and difficulty through the os internum. The presence of clots in the monthly discharge argues the presence of inflammation in the body as well as neck of the organ.

Treatment: Churchill's tincture of iodine to interior of cervix and body of the uterus; cotton ball pessary, saturated with glycerine, left in the vagina till the following day. This was June 22nd. Treatment repeated on the 26th, on July 3rd, 13th, 17th, 23rd, 26th and 30th, and Aug. 8th, 11th, 17th, and 21st. A solution of tannin in glycerine (one part in four) was applied in similar manner on the 24th, 28th, and 31st, and Sept. 7th, 11th, and 18th. Oct. 2nd resumed the tincture of iodine, which was applied Oct. 9th, 12th, 16th, and 23rd—making, in all, twenty-four applications, when the patient was discharged, and was able to pursue her employment through the succeeding autumn and winter with ease and comfort.

Case 3. Mrs. D., native of U. S.; age 32. First menstruated at thirteen years of age—always profuse, and generally painful; twelve years married without conception, although no effort has been made to avoid it. At present, the periods are regular but painful; has gelatinoid leucorrhœa, pain along spinal column, and in pelvis, and often down the course of sciatic and crural nerves; frequent tenderness of back of neck, and neuralgia of the same part. General symptoms of debility, nervous excitability, and cardiac irritability.

Speculum reveals little beyond a gelatiniform discharge from the cervix. Digital examination shows a tender and unduly hard condition of the cervix, with perhaps a very slight degree of prolapsus.

The general treatment adopted was principally mineral acids, with some vegetable bitters and afterwards some chalybeates.

The local treatment adopted was Churchill's tincture of iodine with glycerine, applied to interior of cervix by the application wrapped in cotton, twice weekly, from Aug. 21st to Dec. 1st.

A gradual amelioration of the symptoms enabled this patient to resume the active duties of a housekeeper with entire comfort, at this period.

Case 4. Mrs. S., native of U. S.; age 32; ten years married, has had two children—pregnancy prevented by use of tepid water for several years. Has pain in left hip, ovarian neuralgia, and leucorrhœa. Speculum and sound show superficial ulceration about the os externum, the result of inflammation of the lining of the cervix, which seems to have spread a little from continuity of tissue. Two or three applications of a saturated solution of nitrate of silver, followed by the use of the iodine, the tannin or a weaker solution of the nitrate, twice weekly, from Aug. 24th to Nov. 1st, resulted in so much of improvement that ultimate recovery was certain, when the patient failed to appear at the clinic, having left without being formally discharged.

We now will consider cases (seven in number) where variation from the normal position of the organ in question, is a special symptom, and the result of the same kind of inflammatory process. Of these, the greater part were treated without any mechanical support, and with a view of reducing the inflammatory condition which appeared to be the key to the difficulty. Of the above number five were married and two single. Average time of treatment, three and one-half months; average number of applications, sixteen; average age, twenty-four years.

Case 5. Mrs. H., age 20; of foreign birth; has enjoyed good health till within the past year; complains, at present, of pain in back and in the region of the womb; menstruation regular but painful. Has discharges from the vagina, of thick, tenacious, nearly transparent mucus. Speculum showed cervix turned to the left. The sound developed combined lateral and posterior flexion, considered to be due to a greater degree of inflammatory thickening of the opposite side of the organ, thereby its elongation by this means destroying the symmetry of it.

Has sanguineous discharge following the most careful introduction of the sound into the uterine cavity.

The treatment of this case was the cessation of household work and the use of a thirty-grain solution of nitrate of silver introduced into the cavity of the cervix and body twice each week, with a cotton ball tampon left in the vagina. This method, continued for about twelve weeks, restored the natural position of the organ, and relieved the distress in the pelvis and back. The patient was enabled in much less than the above time to resume her ordinary duties as housekeeper, but continued the applications till recovered.

Especially in a case of long standing where there was decided prolapsus with an inflammatory enlargement, and increased weight of the whole organ, the use of Hodge's closed lever pessary contributed largely to the relief of the symptoms and undoubtedly promoted recovery.

In submitting the foregoing we have endeavored to avoid detail, and arrive at facts and conclusions as far as possible.

We may add that whenever any special symptoms, such as constipation, dyspepsia, neuralgia, palpitation or hæmorrhois, have been present, treatment of general character has been adopted, of which any special notice will hardly be appropriate, as it is foreign to the purpose of this report.

NO. 119 22ND STREET, CHICAGO.

